# 

**DOI:** 10.1192/bjb.2022.27

**Published:** 2023-04

**Authors:** Erin Gourley

**Affiliations:** Core Trainee Year 2 (CT2) in psychiatry with Coventry and Warwickshire Partnership Trust, Coventry, UK. Email: e.gourley@nhs.net


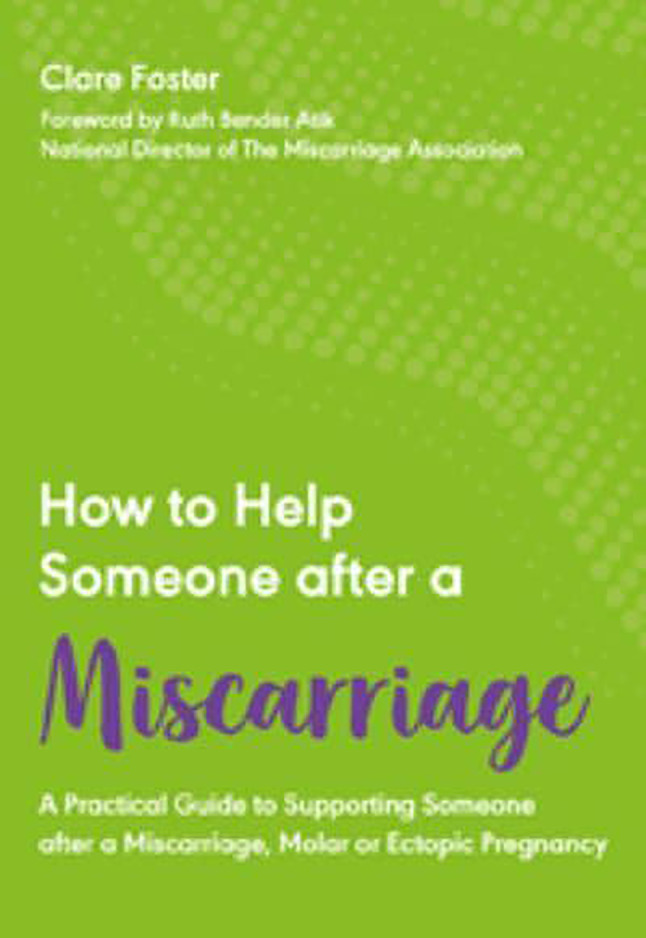


*How to Help Someone after a Miscarriage* aims to advise and support family, friends and partners of people who have suffered any form of pregnancy loss. The author, Clare Foster, is a writer who sadly experienced a miscarriage herself and has since produced supportive resources together with organisations such as Mind and the Miscarriage Association, to name a few.

Foster's own difficult experience combined with her commitment to representing the views of the many women who have experienced miscarriage lends a personal and empathetic quality to the advice provided. The book presents factual summaries of the biology underlying miscarriage, molar and ectopic pregnancy before giving varying accounts from women, partners and friends who have lived experience. A strength of this book is the acknowledgment that processing miscarriage can be different for different people and Foster aims to reflect this through many accounts of others rather than solely relying on her own interpretation. The experiences portrayed in this book are inclusive of different family structures. While acknowledging the impact of pregnancy loss on male partners it also addresses the experiences of female partners; this includes the hurtful assumptions that can sometimes be made and the effect that these can have on their grief. Importantly, the author acknowledges that, despite best intentions, she may not have captured every type of experience.

The book also covers difficult topics such as intimacy problems following loss, recurrent miscarriage and the difficult decision to stop trying to conceive. There is guidance on what to do if you inadvertently say the wrong thing to a grieving partner, relative or friend. The information is presented in a concise and non-judgemental manner, with many suggestions on how best to offer practical and emotional support to someone experiencing pregnancy loss.

I feel that this book achieves its aim and the result is a relatable, digestible and practical resource for supporting women through pregnancy loss. The book is endorsed with a foreword by the National Director of the Miscarriage Association and importantly signposts to many other resources for women and their partners. At a time of great emotional turmoil this book could provide a helpful crutch to family and friends, who otherwise might feel lost, isolated and powerless to help.

